# Dynamic Response of Surface Water Temperature in Urban Lakes under Different Climate Scenarios—A Case Study in Dianchi Lake, China

**DOI:** 10.3390/ijerph191912142

**Published:** 2022-09-25

**Authors:** Haimei Duan, Chunxue Shang, Kun Yang, Yi Luo

**Affiliations:** 1 Faculty of Geography, Yunnan Normal University, Kunming 650500, China; 2 GIS Technology Research Center of Resource and Environment in Western China, Ministry of Education, Yunnan Normal University, Kunming 650500, China; 3 Dean’s Office, Yunnan Normal University, Kunming 650500, China

**Keywords:** climate model, LSWT, Air2water

## Abstract

Lake surface water temperature is a fundamental metabolic indicator of lake ecosystems that affects the exchange of material and energy in lake ecosystems. Estimating and predicting changes in lake surface water temperature is crucial to lake ecosystem research. This study selected Dianchi Lake, a typical urban lake in China, as the research area and used the Air2water model combined with the Mann-Kendall mutation statistical method to analyze the temporal and spatial variation in the surface water temperature of Dianchi Lake under three climate models. The research results show that, under the RCP 5-8.5 scenario model, the surface water temperature change rate for Dianchi Lake from 2015 to 2100 would be 0.28 ℃/10a, which was the largest change rate among the three selected scenarios. The rate of change during 2015–2100 would be 9.33 times higher than that during the historical period (1900–2014) (0.03 °C/10a). Against the background of Niulan River water diversion and rapid urbanization, the surface water temperature of Dianchi Lake experienced abrupt changes in 1992, 2016, 2017, and 2022. Against the background of urbanization, the impact of human activities on the surface water temperature of urban lakes will become greater.

## 1. Introduction

Lake areas account for about 2.8% of the global land surface area and are the hub of the various layers of the earth circle [1], responding to climate change; regulating the climate, water, and land systems [2]; and, with the interaction of the surrounding environment, maintaining the material and energy balance of the ecosystem. They also provide services such as water resources and electricity supply for humans [3] and play an important role in the geosphere [4]. Lake surface water temperature is the most basic physical property of lakes and affects the energy available for lake organisms. In recent years, the global lake surface water temperature has shown an upward trend [5,6], which may potentially affect lake water storage [7], the water cycle [3], lake stratification, and lake physical processes, such as lake responses to climate change [8].

The Coupled Model Intercomparison Project (CMIP) is a fundamental element of climate science [9], and the sixth phase of the CMIP (CMIP6) combines the Shared Socioeconomic Pathway (SSP) [10] and the Representative Concentration Pathway (RCP) [11], providing input data for adaptation to climate change [12] and enabling long-term lake surface water temperature estimates. Most of the current methods for lake surface water temperature estimation are based on machine learning. Yousefi and Toffolon [13] used air temperature as an input variable to predict lake surface water temperature and the results showed that the physical dynamics are still the most important part for the successful prediction of lake surface water temperature [14]. MacCallum predicted lake surface water temperature based on a deep learning (PGDL) hybrid modeling framework [15]. The results showed that the prediction accuracy of the hybrid model was higher than that of the single model, but the energy flow process of the ecosystem was not considered [16]. Piccolroaz developed a mixed physical model (Air2water model) [17] based on the heat transfer equation and regression equation, which, when combined with air temperature data from climate models, could provide a new method for long-term lake surface water temperature prediction [18]. 

Lake surface water temperature estimation is one of the important research areas in the field of lake science. At present, lake surface water temperature prediction is mostly based on deep learning with little consideration of natural physical change processes, and more research is needed for the prediction of lake surface water temperature. Previous research is geographically insufficient, and there are few studies for the surface water temperature of Dianchi Lake. Furthermore, previous research is not sufficient in terms of the time scale, and there are few studies for the prediction of lake surface water temperature across two centuries. Based on the previous research of our team [9,19,20], this article presents a case study conducted at Dianchi Lake by combining the CMIP6 scenario model and using the air temperature as the input parameter in the Air2water model to estimate the surface water temperature of Dianchi Lake. The reproduction of the historical change process for water temperature and the scenario simulation of the future change trend provide basic data for lake ecological environment research and can be used as a reference for lake water environment management.

## 2. Study Area and Methods

### 2.1. Study Area

**Figure 1:**
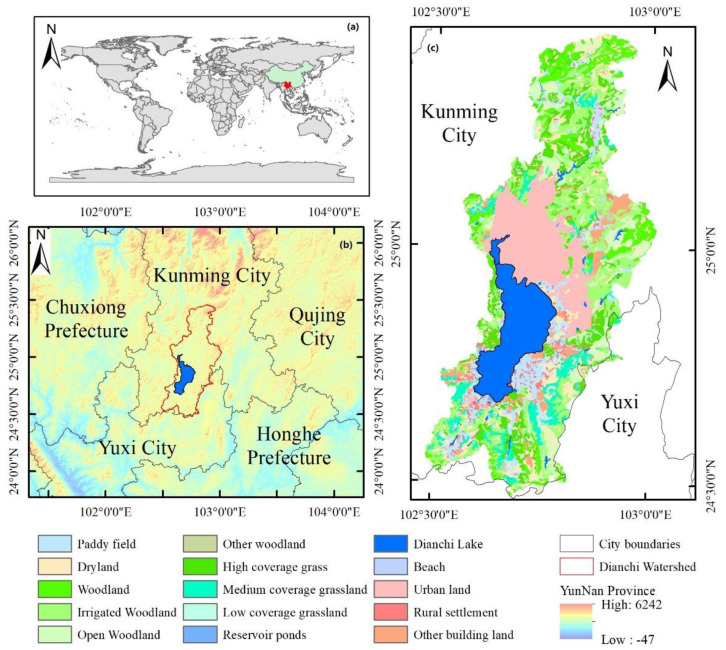
Overview of the study area: (**a**) shows Yunnan Province from a global perspective, (**b**) shows the DEM topographic information of the Dianchi Lake Basin, and (**c**) shows the land-use information for the Dianchi Lake Basin in 2018. A positive sign indicates a positive value above the earth level in (**b**) and a negative value below.

Dianchi Lake (Figure 1) is located southwest of Kunming City, Yunnan Province. It is one of the famous freshwater lakes in Yunnan Province and a representative urban lake in China [21]. Dianchi Lake belongs to the Jinsha River system in the Yangtze River Basin and is a typical lake in terms of lake management in China. The lake is a rifted structural lake, with a catchment area of 2866.0 km^2^ and a lake recharge coefficient of 9.6. There are more than 20 large and small rivers flowing into Dianchi Lake, among which the Panlong River, Ma Liu River, Luolong River, and Liangwang River constitute a total catchment area of more than 100 km^2^ on the east, south, and north sides respectively [22]. The Haikou on the southwest side of the lake is the only outlet of the Dianchi Lake. It flows into the Jinsha River via the Tanglang River and the Pudu River. The Haikou has a gate to regulate the storage and discharge of the lake. When the water level is 1887.5 m, the water area of Dianchi Lake is 300.4 km^2^, the maximum water depth is 9.9 m, the average water depth is 5.4 m, and the water storage capacity is 16.0 × 10^8^ m^3^. The lake surface water temperature affects energy dynamics for the lake ecosystem and is closely related to the lake’s aquatic organisms and water quality. The estimation of the surface water temperature of Dianchi Lake is very important for the lake’s ecological environment and has implications for lake water pollution control. The study showed that the annual precipitation in the Dianchi basin demonstrated an overall non-significant decreasing trend, while the annual average temperature and the annual average maximum (low) temperature showed significant increasing trends; the descending order for the percentage variation in precipitation in the basin in different seasons was winter > summer > spring > autumn; the annual average temperature, maximum temperature, and minimum temperature in Dianchi basin showed increasing trends, and the descending order for the increase in temperature in the basin in different seasons was spring > summer > autumn > winter. In 2000, the water quality of Dianchi Lake changed from Class III to Class V and the impervious coverage rate reached 21.56%. From an economical perspective, the Dianchi Lake facilitates various economic functions, such as regulating water resources, multiplying aquatic products, and allowing navigation [23] to major domestic and foreign tourist attractions, which are important tourism resources in Yunnan Province.

### 2.2. Research Data

Table 1 presents the databases used in this study. This study used the air temperature data from 1900 to 2100, and the lake surface water temperature data from MODIS 11A2 (Terra) from 2001 to 2021 as basic data. The air temperature data were input into the model to estimate the lake surface water temperature after various preprocessing steps, such as format conversion, clipping, and extraction of information values. The MODIS data were used to verify the water temperature data estimated by the model after format conversion, splicing, clipping, and other preprocessing steps. This study used Landsat8 OLI 2001–2021 image data, a DEM (SRTM30 m), and China’s basic geographic boundary information as auxiliary data. The Landsat8 OLI data were extracted from lake boundary data based on Google Earth Engine, and the DEM data were extracted from lake watershed data. The basic information on the data used is shown in Table 1.

Air temperature data (tas) in scenario mode were downloaded from the CMIP6 website, and the spatial resolution of the downloaded data was 100 km. The data were resampled (to 10 km), reprojected, and cropped after being extracted from the air temperature data based on the ENVI 5.3 IDL8.5 platform (Yunnan Normal University, Kunming, China) to predict water temperature. The lake boundaries used for clipping were extracted from the Google Earth Engine platform using Landsat8 OLI data.

### 2.3. Method

#### 2.3.1. Air2water Model

The basic concept of the Air2water model [18] is based on the hypotheses that air temperature is an external integrated effect of water temperature increase, that it controls the surface heat balance of lakes, and that water temperature fluctuations depend directly on the heat flux in the surface water and the surface area of the lake [24], the volume of surface water of the lake involved in heat exchange (reaction volume). The total heat integral heat equation for the reaction volume is shown in Equation (1). The relationship between the air temperature ($T_{a}$) and the lake water temperature ($T_{w}$) in the lake surface water heat flux was obtained with the linear Taylor expansion (Equation (2)).

(1) $$\rho C_{p}V_{s}\frac{dT_{w}}{d_{t}}=AH_{net}$$

(2) $$H_{net}\approx H_{net,0}+\frac{\partial H_{net}}{\partial T_{a}}|\bar{T_{a}},\bar{T_{w}}(T_{w}-\bar{T_{w}})$$

Here [25], $T_{w}$ represents the water temperature, t represents time, *H_net_* represents the heat flux of the surface water, A represents the lake surface area, $V_{s}$ represents the volume participating in atmospheric heat exchange, ρ represents the lake density, and $C_{p}$ represents the lake constant specific heat capacity. The net heat flux per unit surface $H_{net}$ at the air–water interface can be calculated as shown in Equation (3):

(3) $$H_{net}=H_{s}+H_{a}+H_{w}+H_{e}+H_{c}+H_{p}+H_{i}+H_{d}$$

where $H_{w}$ is the net longwave radiation emitted from the lake; $H_{a}$ represents the net longwave radiation emitted by the atmosphere and absorbed by the lake; $H_{e}$ is the latent heat flux due to evaporation and condensation; $H_{c}$ represents the sensible heat flux due to convection; $H_{p}$ represents the heat flux due to incoming precipitation; $H_{i}$ represents the heat exchanged with inlets/outlets; and $H_{d}$ represents the heat exchanged between the surface volume and the deep water or sediment.

The Air2water model is a hybrid model based on physical statistics, the fundamental principle of which is to use the fundamental law of energy transfer from physics to achieve an estimate of the surface water temperature of a lake when only the air temperature is known. The Air2water model is a valuable alternative tool to simpler regression models, although it cannot take into account the hysteresis cycle between air and water temperatures. In this study, we used Air2water to estimate [17] the surface water temperature of the lake and investigate the response of the surface water temperature of Dianchi Lake to air temperature changes under climate change scenarios.

#### 2.3.2. Three Climate Models

The Scenario Model Intercomparison Project (ScenarioMIP), the main activity of Phase VI of the Coupled Model Intercomparison Project (CMIP6) [26], can play a role in improving the understanding of the climate system and characterizing societal risks and coping options. The project can play an important role in providing multi-model climate projections of future emissions and land-use changes based on alternative scenarios generated by integrated assessment models. These climate projections are driven by a new set of emissions and land-use scenarios [27] using an integrated assessment model (IAM) based on new future social development pathways, shared socioeconomic pathways (SSPs), and associated representative concentration pathways (RCPs). CMIP6 climate projections differ from those in CMIP5 because they are not only generated using an updated version of the climate model but are based on updated data on recent emissions trends and driven by SSP-based scenarios generated using an updated version of the IAM [28]. Based on ScenarioMIP, we took into account future climate change, on the one hand, and land use, GDP, and other human activities, on the other hand, using the scenario model air temperature data (Tas) provided by CMIP6 and inputting it into the Air2water model after preprocessing to estimate the surface water temperature of Dianchi Lake. The objective of this study was to explore the changing behavior of the surface water temperature of Dianchi Lake in the future climate under three scenarios (high, medium, and low) and, thus, provide a theoretical basis for water body management in Dianchi Lake.

#### 2.3.3. The Mann–Kendall Test

The Mann–Kendall Test is a commonly used statistical test with the advantages of not requiring that samples follow a certain distribution and not being disturbed by a few outliers, as well as being relatively widely applicable easy to calculate. The principle of the Mann–Kendall test is that, for a time series X (containing *n* samples), an order column is constructed (as shown in Equation (4) and the statistics are defined, as shown in Equation (5):

(4) $$s_{k}=\sum_{i=1}^{k}r_{i}\;(k=2,3,\ldots ,n)$$

$$r_{i}=\left\{ \begin{array}{l} +1\;\;x_{i}>x_{j}\;(j=1,2,\ldots ,i)\\ 0\;\;\;else \end{array}\right.$$

(5) $$UF_{k}=\frac{\left[s_{k}-E(s_{k})\right]}{\sqrt{Var(s_{k})}}\;(k=1,2,\ldots ,n)$$

where $UF_{k}=0,E(s_{k})$ is the mean of $s_{k}$ and $Var(s_{k})$ is the variance of $s_{k}$. Then, in the inverse order of the time series X, when the above process is repeated, $UB_{k}=UF_{k}\;(k=n,n-1,\ldots ,1)$, $UB_{1}=0$. Our surface water temperature characterization of Dianchi employed trend analysis and feature analysis, and we used the Mann–Kendall Test to reveal the sudden change characteristics in Dianchi surface water temperature. We chose the significance level α=0.05, with a critical value of $U_{0.05}=\pm 1.96$. Values of $UF_{k}$ and $UB_{k}$ greater than 0 indicate significant upward or downward trends, while values lower than 0 indicate a downward trend; when they exceed the critical line, they indicate a significant upward or downward trend, and the range above the critical line is determined as the time region where the mutation occurs. If the two curves of $UF_{k}$ and $UB_{k}$ intersect and the intersection point is between the critical lines, then the moment corresponding to the intersection point is the time when the mutation starts [29].

## 3. Results and Discussion

### 3.1. Characteristics Analysis of Surface Water Temperature Changes in Dianchi under Three Scenarios

**Figure 2:**
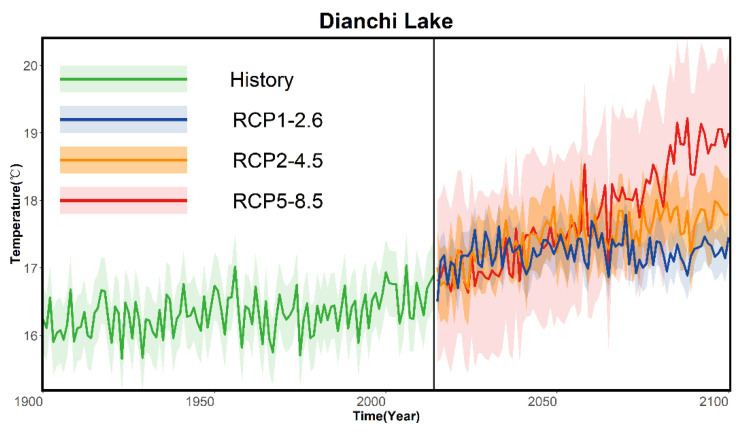
Trend diagrams for the annual average lake surface water temperature in Dianchi Lake under the three scenarios. The thick line represents the average annual water temperature, the legend shows the model information, and the shading represents the ±1.64 σ interval, where σ is the standard deviation of the annual average (close to the average). The research results show that the surface water temperature of Dianchi Lake did not change much in the historical period; the surface water temperature of Dianchi Lake shows different variation characteristics in different scenarios.

The data series were divided into four feature sets, as shown in Figure 2: the historical change feature set (1900–2014), the RCP1-2.6 model feature set (2015–2100), the RCP2-4.5 model feature set (2015–2100), and the RCP5-8.5 (2015–2100) model feature set. The annual rate of change in the surface water temperature in Dianchi from 1900–2014 was 0.03 °C/10a; the rate of change in the surface water temperature in Dianchi for RCP1-2.6 (1900–2100) was projected to be 0.05 °C/10a; the rate of change in the surface water temperature in Dianchi for RCP2-4.5 (1900–2100) was projected to be 0.10 °C/10a; the rate of change in the surface water temperature in Dianchi for RCP5-8.5 (1900–2100) was projected to be 0.28 ℃/10a. The change rate (0.28 °C/10a) for the RCP5-8.5 scenario model (2015–2100) was the greatest, and the change rate (0.03 °C/10a) for the historical period (1900–2015) was the smallest. Compared with the historical period (1900–2015), the surface water temperature change rate in Dianchi Lake in the RCP1-2.6 scenario model (2015–2100) was increased by 0.66. Compared with RCP1-2.6 (2015–2100), the rate of change doubled; the rate of change in the surface water temperature in Dianchi Lake was 1.8 times greater compared to RCP2-4.5 (2015–2100) in the RCP5-8.5 scenario model (2015–2100). Under the scenario model RCP5-8.5 (2015–2100), the growth rate for the surface water temperature in Dianchi Lake in the RCP5-8.5 scenario model (2015–2100) was 9.33 times that of the historical stage (1900–2014).

**Figure 3:**
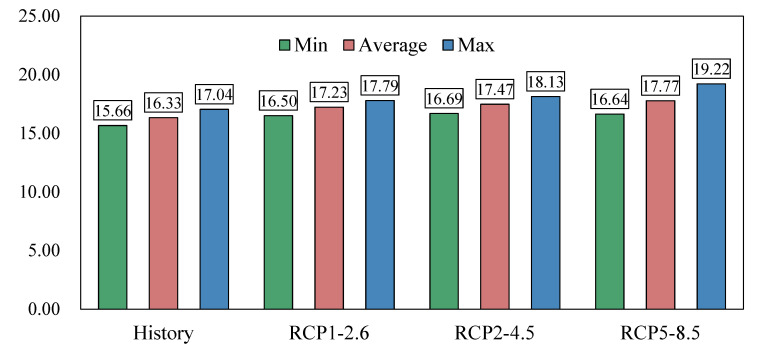
Average and extreme values of surface water temperature in Dianchi Lake.

As shown in Figure 3, in the historical stage, the average surface water temperature of Dianchi Lake was 16.33 °C, the maximum value was 17.04 °C (2006), and the minimum value was 15.66 °C (1923). The temperature reached a maximum of 17.04 °C, which is consistent with the findings of previous studies [8], and the water quality in Dianchi was generally worse than Class V in 2006, suggesting that an excessively warm climate leads to higher water temperatures, which translates into a deterioration of water quality (faster mineralization of substances in water and sediments and more nutrients available to primary producers). Under the scenario model RCP1-2.6, the future average surface water temperature of Dianchi Lake will be 17.23; the maximum lake surface water temperature will be 17.79, which will appear in 2070; and the minimum surface water temperature of Dianchi Lake was 16.50 (2015). Under the scenario model RCP2-4.5, the mean value of the lake surface water temperature is 17.47 °C, reaching a maximum value of 18.13 °C in 2057 and a minimum value of 16.69 °C in 2024. Under the scenario model RCP5-8.5, the average value of the lake surface water temperature is 17.77 °C, and Dianchi Lake surface water temperature will reach a minimum value in 2024 (16.64 °C), and the maximum value will appear in 2088 (19.22 °C). Under the three scenario models, the future surface water temperature of Dianchi Lake shows high-temperature scenarios corresponding to a high rate of change and a high lake surface water temperature value, indicating that under the scenario of climate change and intensified human activities [30], the lake surface water temperature will undergo a warming effect. Climate changes from stronger disturbances from human activities will result in higher lake surface water temperatures, which is consistent with the conclusion drawn in [31].

### 3.2. Analysis of Abrupt Changes in the Annual Average Lake Surface Water Temperature of Dianchi Lake under the Three Scenarios

**Figure 4:**
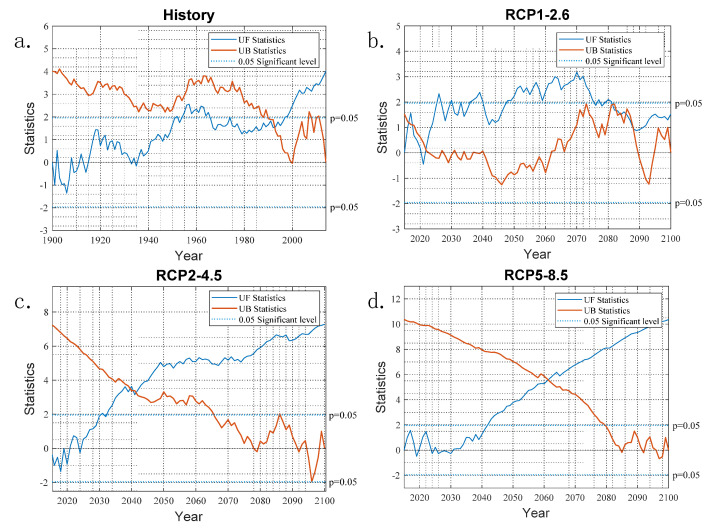
Dianchi Lake MK mutation test, where (**a**) represents the result of the MK mutation test for the lake surface water temperature in the historical period (1990–2014), (**b**) represents the lake surface water temperature MK mutation test result under the scenario model RCP1-2.6 (2015–2100), (**c**) indicates the MK mutation test result for the lake surface water temperature under the scenario model RCP2-4.5 (2015–2100), and (**d**) represents the MK mutation test result for the lake surface water temperature under the scenario model RCP5-8.5 (2015–2100).

The Mann–Kendal test results for the surface water temperature of Dianchi Lake are shown in Figure 4. In the historical time period, it can be seen from the UF curve that the value of the UF in the 15 years from 1900 to 1915 was less than 0, except in 1908 and 1912, showing a clear downward trend. If it is less than 0, it indicates an obvious downward trend, and the UF values in other years are all greater than 0, showing an obvious upward trend. The intersection of the UF line and the UB line in the figure is in 1992, indicating that the change in trend for the surface water temperature of Dianchi Lake in the historical stage occurred in 1992, and it was a sudden change. The main reason for this was that the water in Dianchi Lake became Class V water in 1990 [23,32]. At the same time, the population of the Dianchi Lake basin increased rapidly in 1992, which increased the demand for industrial and domestic water and increased the water quality and human activities. Under this dual pressure, a sudden change in the surface water temperature of Dianchi Lake appeared in 1992, which is consistent with the conclusions of previous studies [33].

The surface water temperature of Dianchi Lake was analyzed under the scenario model RCP1-2.6. It can be seen that the UF line was greater than 0 in all years except 2021, showing an obvious upward trend; the intersection points of the UF line and the UB line were in 2016, 2017, and 2022, indicating that the surface water temperature of Dianchi Lake changed in 2022 under the scenario model RCP1-2.6, and it was a sudden change [34]. The years 2016 and 2015 showed positive mutations, and 2022 showed a negative mutation. Under the scenario model RCP1-2.6, the surface water temperature of Dianchi Lake showed three sudden changes in 2016, 2017, and 2022. The reason for these changes is that the Niulanjiang–Dianchi Lake water replenishment project was implemented at the end of 2013, and the drinking water project was implemented from 2014 to 2018. This shows that the drinking water project disturbed the original trend in the surface water temperature of Dianchi Lake [19]. At the same time, the implementation of the urban development strategy of “one lake and four areas, extending from the south to the north” in the Dianchi Lake basin will affect the surface water temperature of Dianchi Lake. Later team research will focus on analyzing the relationship between lake surface water temperature and water quality, biomass, etc., under climate change.

In the Mann–Kendal test results for the surface water temperature of Dianchi Lake under the scenario model RCP2-4.5, the UF line values for all years except for 2015, 2016, 2017, 2018, 2019, 2020, and 2024 were greater than 0, indicating an obvious upward trend, but the intersection of the UF line and the UB line was not within the test range, indicating that the surface water temperature parameters of Dianchi Lake under this scenario model did not change abruptly. Except for the years 2019, 2024, 2026, 2027, 2028, 2029, and 2030, the UF line values of all the other years were greater than 0 in the Mann–Kendal test results for the surface water temperature of Dianchi Lake under the scenario model RCP5-8.5, showing that there was an obvious upward trend, but the intersection of the UF line and the UB line was not within the test range, indicating that the surface water temperature parameters of Dianchi Lake under this scenario model were not subject to abrupt changes. This shows that the urban Dianchi Lake best matched with the scenario model RCP1-2.6 among the three selected scenarios (RCP1-2.6, RCP2-4.5, and RCP5-8.5).

### 3.3. Influence of Lake Surface Water Temperature Changes on Lake Ecological Environment under Three Scenarios

Under the scenario model RCP1-2.6, the growth rate for the surface water temperature of Dianchi Lake was 0.05 ℃/10a; under the scenario model RCP2-4.5, the growth rate of the surface water temperature of Dianchi Lake was 0.10 ℃/10a; under the scenario model RCP5-8.5, the growth rate of the surface water temperature of Dianchi Lake was 0.28 °C/10a. The growth rate of the surface water temperature in Dianchi Lake in the low-emission scenario (RCP1-2.6) was lower than that in the high-emission scenario (RCP5-8.5). The increase in the surface water temperature of Dianchi Lake will promote harmful cyanobacterial blooms in Dianchi Lake [6]. Studies have shown that the initiation of cyanobacterial blooms in lakes is related to temperature. For example, in the subtropical region of China, Taihu Lake cyanobacteria blooms mostly occur in warm waters in spring and Microcystis blooms occur in earlier spring and warmer water bodies [35]. Cyanobacterial blooms will result in lower concentrations of dissolved oxygen, which is required by aquatic organisms [36]. When the water temperature rises and the dissolved oxygen concentration decreases, extensive deoxygenation patterns will appear in Dianchi Lake, aggravating the severity of deoxygenation caused by the eutrophication of the lake and resulting in the death of fish and birds [3], and the deterioration of water quality will be more severe [3] and pose a serious threat to human health in the lake basin [28].

### 3.4. Water Temperature Indication of RCP5-8.5 Scenario Model

The maximum value of the surface water temperature of Dianchi under the future scenario model RCP5-8.5 (2015–2100) would be 19.22 °C, the average water temperature would be 16.64 °C, and the minimum water temperature would be 17.77 °C. The water temperature growth rate would be 9.33 times higher than that of the historical period (1900–2014); this result may be due to the global climate change in recent years [5], as well as the effects of global warming, exacerbated by increased greenhouse gas emissions due to human activities, such as urbanization [20], which trigger changes in the lake basin climate and indirectly lead to an increase in lake surface water temperature [33]. These results suggest that human activities, such as those resulting in climate change, can have an impact on the surface water temperature of Dianchi, and this impact can interfere with the thermal balance of the lake system itself, affecting the species diversity of the lake ecosystem, the water quality, and human life in the basin. Therefore, it is crucial to develop sustainable approaches to energy change and energy conservation to avoid generating RCP5-8.5 scenario patterns.

## 4. Conclusions

Based on the climate change scenario model in combination with the Air2water model used to estimate the lake surface water temperature in Dianchi Lake from 1900 to 2100, the following conclusions can be drawn: (1) The surface water temperature of Dianchi Lake under different scenario models will show different variation characteristics. Under SSP5-RCP5-8.5, the surface water temperature change rate for Dianchi Lake will be the greatest, 9.33 times that of the lake surface water temperature change rate in the historical period. (2) Human activities have an impact on the sudden change in surface water temperature in Dianchi Lake. In 1990, when the population around the Dianchi Lake increased and the water in the lake became Class IV water, the surface water temperature showed abrupt changes in 1992. From 2014 to 2018, during the Niulan River drinking water project, there were two abrupt changes in the surface water temperature in Dianchi Lake. Therefore, it is evident that, when the surrounding areas of the lake became densely populated and urbanized, the deterioration in water quality had an impact on the surface water temperature of the lake.

## Figures and Tables

**Table 1:** Research data information.

Name	Dataset Source	Download URL
LSWT	MOD11A2	https://ladsweb.modaps.eosdis.nasa.gov/ (accessed on 23 April 2022)
Tas	ERA-Interim	https://esgf-node.llnl.gov/search/cmip6/ (accessed on 30 June 2022)
Lake boundaries	Landsat8 OLI	https://glovis.usgs.gov (accessed on 15 June 2022)
Watershed	DEM(SRTM)	https://glovis.usgs.gov (accessed on 10 June 2022)
Base boundaries	China’s administrative division data	http://www.ngcc.cn/ngcc/ (accessed on 5 May 2022)
LUCC	CNLUCC	http://www.redc.cn (accessed on 12 February 2022)

## Data Availability

The data that support the findings of this study are available from the corresponding author upon reasonable request.
